# The Antitumor Effects of Plasma-Activated Saline on Muscle-Invasive Bladder Cancer Cells In Vitro and In Vivo Demonstrate Its Feasibility as a Potential Therapeutic Approach

**DOI:** 10.3390/cancers13051042

**Published:** 2021-03-02

**Authors:** Hao Zhang, Jishen Zhang, Bo Guo, Hailan Chen, Dehui Xu, Michael G. Kong

**Affiliations:** 1 State Key Laboratory of Electrical Insulation and Power Equipment, Centre for Plasma Biomedicine, Xi’an Jiaotong University, Xi’an 710049, China; zhang216@mail.xjtu.edu.cn (H.Z.); zjsxjtu@163.com (J.Z.); bo_guo@xjtu.edu.cn (B.G.); 2 Frank Reidy Center for Bioelectrics, Old Dominion University, Norfolk, VA 23508, USA; h1chen@odu.edu; 3 Department of Electrical and Computer Engineering, Old Dominion University, Norfolk, VA 23529, USA

**Keywords:** muscle-invasive bladder cancer, cold atmospheric plasma, plasma-activated saline, reactive oxygen species, intravesical perfusion

## Abstract

**Simple Summary:**

Bladder cancer is the most common urinary system carcinoma, with more than 430,000 new cases diagnosed every year. More than 25% of patients are classed as having muscle-invasive bladder cancer (MIBC). MIBC is a serious clinical problem and is fatal for the majority of patients. In this work, we focus on the feasibility of plasma-activated saline (PAS) as a potential therapeutic approach for the treatment of MIBC. For this purpose, we evaluated the anticancer effect of PAS on two human bladder cancer cell lines (T24 and J82) in vitro and in vivo. Our initial results demonstrated that the PAS can provide a novel and valuable therapeutic effect for the local treatment of MIBC. We believe that the results presented in this paper will be of interest to many scientists in the field of bladder cancer treatment and plasma biomedicine.

**Abstract:**

Muscle-invasive bladder cancer (MIBC) is a fast-growing and aggressive malignant tumor in urinary system. Since chemotherapy and immunotherapy are only useable with a few MIBC patients, the clinical treatment of MIBC still faces challenges. Here, we examined the feasibility of plasma-activated saline (PAS) as a fledgling therapeutic strategy for MIBC treatment. Our data showed that plasma irradiation could generate a variety of reactive oxygen species (ROS) and reactive nitrogen species (RNS) in saline. In vivo tests revealed that pericarcinomatous tissue injection with PAS was effective at preventing subcutaneous bladder tumor growth, with no side effects to the visceral organs after long-term administration, as well as having no obvious influence on the various biochemistry indices of the blood in mice. The in vitro studies indicated that adding 30% PAS in cell culture media causes oxidative damage to the bladder transitional cells T24 and J82 through enhancing the intracellular ROS level, and eventually induces cancer cells’ apoptosis by activating the ROS-mediated Fas/CD95 pathway. Therefore, for an intracavity tumor, these initial observations suggest that the soaking of the tumor tissue with PAS by intravesical perfusion may be a novel treatment option for bladder cancer.

## 1. Introduction

Bladder cancer is the most common malignant tumor of the genitourinary system, with a high incidence and recurrence rate in males. Almost 90% of bladder cancers are transitional cell carcinoma [1,2]. Nowadays, surgery, radiotherapy, chemotherapy, and biotherapy are the conventional treatments for patients with bladder cancer. In numerous clinical procedures, these treatments are used in conjunction with each other to improve local therapeutic efficacy and prevent the development of distant metastases [3]. Nonmuscle invasive bladder cancer is treated with transurethral bladder tumor resection and subsequent pelvic radiotherapy or intravesical instillation chemotherapy, as well as the bacillus calmette-guerin (BCG) vaccine [4]. Despite the therapeutic strategy usually being conservative, the clinical therapeutic effects for nonmuscle invasive bladder cancer are acceptable. For treatment of muscle-invasive bladder cancer (MIBC), surgical removal and chemotherapy or immunotherapy are preferred because of the inefficiency of radiotherapy [5]. However, on account of chemotherapy only being applied for about 50% of MIBC patients due to serious side effects, and the latest immune checkpoint blockade therapy only really working in 15–25% of MIBC patients, the clinical treatment of MIBC still faces challenges. A lack of effective medical therapeutic measures have led to a low five-year survival rate of MIBC (about 35% of nonmetastatic MIBC and only 6% of metastatic MIBC) [6]. Thus, new therapies are needed to improve treatments for MIBC.

Cold atmospheric plasma (CAP) is an ionized gas generated by electrical discharges at room temperature in atmospheric pressure [7]. It is a gaseous mixture consisting of positive/negative ions, radicals, electrons, neural atoms, excited molecules, ultraviolet photons (UV), and a transient electric field. As it causes no thermal damage to heat-sensitive material or living cells and tissues, CAP has emerged as an alternative approach in medical applications such as the sterilization of medical devices, blood coagulation, root canal therapy, wound healing, tissue regeneration, and cancer treatment [8,9,10,11]. CAP generates reactive oxygen species (ROS) and reactive nitrogen species (RNS), which are considered to play a crucial role in these applications [12,13,14,15,16,17,18]. Many researchers have shown that a low dose of plasma irradiation can promote cell proliferation, whereas a high dose of plasma irradiation can inhibit cell proliferation and even induce programmed cell death [19,20,21]. Significantly, recent in vitro studies of CAP for cancer treatment have demonstrated that suitable dosages of plasma irradiation can selectively kill multiple types of cancer cells, and even drug-resistant cancer cells, while causing little injury to normal tissue cells [22,23,24]. In addition, several in vivo experiments have reported that plasma direct irradiation reduces subcutaneously implanted tumors in nude mice from glioblastoma, ovarian cancer, and vestibular schwannoma [12,24,25]. In clinical research, CAP irradiation has also been verified as an effective and safe intervention to treat advanced head and neck cancer [26,27]. As a consequence, CAP has been studied as a new therapeutic strategy for cancer, and the interest in CAP’s anticancer effects is growing.

Despite the promising anticancer effects on advanced head and neck cancer that have already been demonstrated, the clinical adoption of direct CAP irradiation is currently limited to skin cancer and exposed tumor tissues. With the currently available technology, it is difficult to import the plasma into the bladder for MIBC treatment. However, recent studies have indicated that a cell culture medium or other liquid irradiated by CAP could also induce cancer cells apoptosis in vitro and in vivo [28,29,30,31,32]. Plasma-irradiated solutions with anticancer effects are termed plasma-activated solutions [33]. It is considered that, during CAP irradiation, the solutions were enriched with a wide variety of aqueous ROS and RNS, such as hydrogen peroxide (H_2_O_2_), hydroxide radicals (OH•), superoxide anion radicals (O_2_^−^), ozone (O_3_), nitrite ions (NO_2_^−^), peroxynitrite (ONOOH/ONOO^−^), and nitrate ions (NO_3_^−^) [33,34,35,36,37,38,39]. These aqueous ROS and RNS can trigger intracellular oxidative stress, mitochondrial dysfunction and DNA damage, and further activate the related signaling pathways of programmed cell death [23,40,41,42,43]. Moreover, it is worth noting that the plasma-activated solutions have the potential to greatly facilitate the clinical applications of CAP, because plasma-activated solutions are likely to break through the treatment-depth limitations of direct CAP irradiation. Plasma-activated solutions can be used as a medicament by injecting them into or near the site of deeper tumors, or perfusing them into the bladder and abdominal cavity, which are hard to reach for direct CAP irradiation. Hence, plasma-activated solution injection or lavage is expected to be an alternative therapy pathway for deep tumors and intracavity tumors. Plasma-activated solutions might help to overcome multidrug resistance and further upgrade cancer therapy. However, while the action depth of plasma-activated solutions for cancer therapy is superior to CAP direct treatment, the antitumor effects of plasma-activated solutions for MIBC treatment are unknown, and its biosafety still needs more investigation.

As an intracavity tumor, the paracancerous injection or intravesical perfusion of plasma-activated solutions for the local infusion treatment of MIBC may be a potential therapeutic approach, and more acceptable clinically. In this study, we evaluated the antitumor effects of surface discharge plasma-activated saline (PAS) on muscle-invasive human bladder cancer cells (transitional bladder cancer cell lines: T24 and J82 cells) in a xenograft model, as well as the underlying mechanisms and possible toxicity. The objective of this study was to verify the potential of PAS infusion as a novel treatment strategy for muscle-invasive bladder cancer.

## 2. Materials and Methods

### 2.1. Experimental Device Configuration

**Figure 1:**
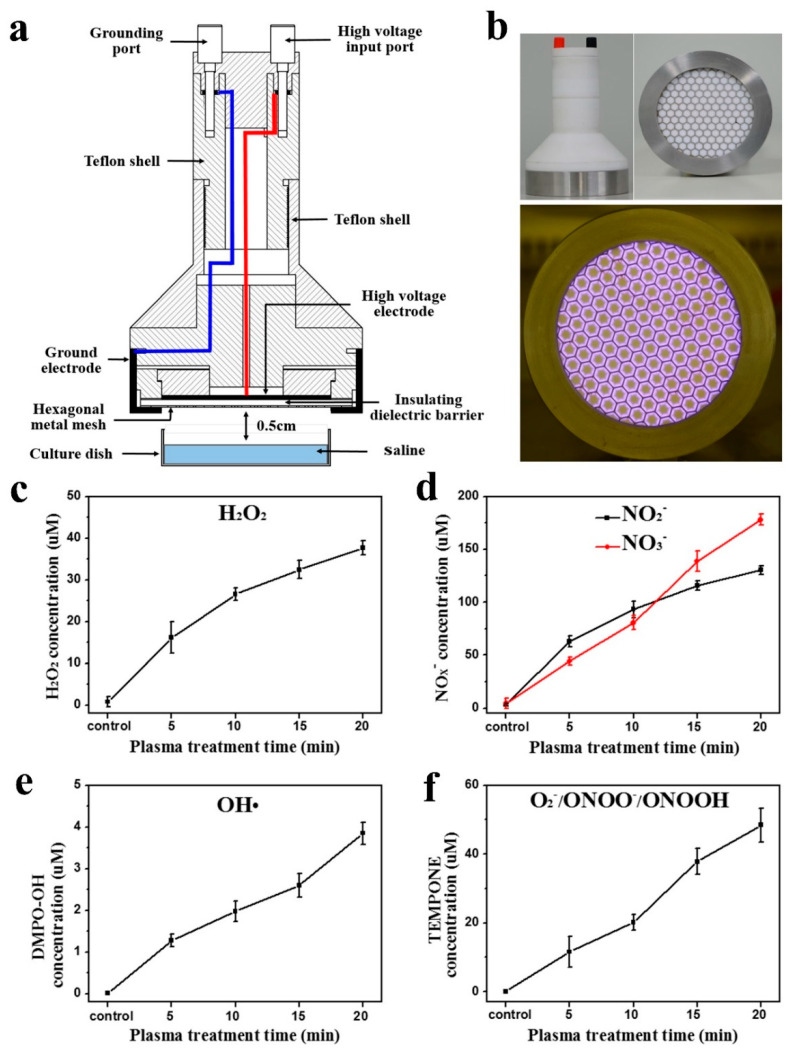
(**a**) Schematic diagram of the cold atmosphere plasma generation system; (**b**) photograph of the plasma device and plasma discharge. Reactive species level in the saline after 20 min plasma treatment: (**c**) H_2_O_2_; (**d**) NO_x_^−^; (**e**) OH•; (**f**) O_2_^−^ and ONOO^−^/ONOOH (*n* = 3).

The experimental setup consists of a surface discharge reactor and a downstream culture dish of saline, as shown in Figure 1a. The discharge reactor is composed of a plane HV electrode, a grounded mesh electrode, and a dielectric sheet sandwiched between the two electrodes. The HV electrode and ground electrode are connected to the high voltage input port and grounding port, respectively, through wires surrounded by a Teflon shell. As shown in Figure 1b, each grounded mesh has a hexagon shape and supports a microdischarge. Details of the discharge reactor have been reported in our previous publications [44]. The distance between the grounded metal mesh and the saline is 0.5 cm. The applied high voltage is a sinusoidal, with a peak-to-peak value of 8 kV at a frequency of 10 kHz, the discharge power is 950 mW, and the power density is 0.02 W·cm^−2^. The waveform of discharge voltage is recorded by an oscilloscope (Tektronix, DPO3000, Beaverton, OR, USA) with a high-voltage probe (Tektronix, P6015A). In this study, 5 mL saline was placed in a 60 mm dish and activated by plasma for 20 min at room temperature. After 20 min discharge, the electrodes’ temperature increased from 24.9 ± 0.7 °C to 29.6 ± 2.3 °C, and the temperature of the saline was essentially unchanged.

### 2.2. Measurements of Aqueous ROS and RNS

Here, aqueous H_2_O_2_ was measured using the Amplex Red Hydrogen Peroxide/Peroxidase Assay kits (Beyotime, Shanghai, China) according to the manufacturer’s instructions. Aqueous NO_2_^−^ and NO_3_^−^ were measured using the Griess reagent kit (Beyotime, Shanghai, China). Aqueous OH• was detected using the DMPO (5,5-dimethyl-1-pyrrolineN-oxide, Dojindo, 5 mM) by an electron spin resonance (ESR) spectrometer (BrukerBioSpin GmbH, EMX, Ettlingen, Germany). The contents of aqueous O_2_^−^/ONOO^−^/ONOOH were measured using the TEMPONE-H (1-hydroxy-2,2,6,6-tetramethyl-4-oxo-piper-idine, Enzo, 5 mM). Detailed ROS and RNS detection methods are outlined in our previous paper [45]. PAS sampling for H_2_O_2_, NO_2_^−^ and NO_3_^−^ measurement were completed within 5 min after plasma irradiation due to other operations. The DMPO (or TEMPONE-H) were added into the saline before plasma irradiation, and also within 5 min after irradiation the PAS sampling (containing DMPO or TEMPONE-H) was analyzed by ESR. All experiments were performed three times (*n* = 3).

### 2.3. Cell Culture

Invasive bladder cancer cell lines T24 and J82 were sourced from the Cell Bank of the Chinese Academy of Sciences (Shanghai, China). Both the T24 and J82 cell lines were cultured in complete medium, containing Dulbecco’s modified Eagles medium, (DMEM, Invitrogen Life Technologies, Carlsbad, CA, USA), 10% (*v/v*) fetal bovine serum (Gibco, Grand Island, NY, USA), and 1% (*v/v*) penicillin-streptomycin solution (Beyotime, Shanghai, China), at 37 °C in 5% CO_2_.

### 2.4. Animal Model and Treatment

Twenty-five BALB/c male mice aged eight weeks, with an average weight of 24 ± 3 g, were obtained from the Experimental Animal Center of Xi’an Jiaotong University, Xi’an, China. All the mice were raised in a specific pathogen free class of housing in the laboratory. The twenty-five mice were randomly divided into three groups: (I) control group— five mice were given no cells and liquid injection (*n* = 5); (II) T24 tumor group—ten mice were used for tumor formation with T24 cells; (III) J82 tumor group—ten mice were used for tumor formation with J82 cells. T24 cells (1 × 10^6^ in 100 µL PBS) or J82 cells (1 × 10^6^ in 100 µL PBS) were injected subcutaneously into the left rear flank of each mouse [46]. After fourteen days of tumor formation, tumor-bearing mice were randomly divided into the saline-treatment and PAS-treatment groups (each group consisted of five mice, *n* = 5). Then, the tumor-bearing mice were administered pericarcinomatous tissue injections with saline or PAS (the interval time between generating the PAS and injection in vivo is 1 h) at the dosage of 200 µL [24,47]. In short, a small syringe (loaded with 200 µL saline or PAS) was used to gently stir up the skin above the tumor and then inject saline or PAS. After injection, 200 µL saline or PAS would form a droplet at the subcutaneous site near the tumor. The saline or PAS injection was repeated three times a week [24]. The growth of tumors was monitored by Vernier caliper measurements of the two perpendicular diameters, and the tumor volume was calculated with the formula V = 0.52 × (width^2^ × length) [48,49]. For the in vivo fluorescence imaging, the mice were administered 1.5 mg D-Luciferin before isoflurane anesthesia. Bioluminescent images were acquired using a macroillumination imaging system and tunable lighting system (Optonics, Lexington, KY, USA). The survival time of each mouse was recorded. When the tumor-bearing mice suffered behavior problems (abnormal feeding behavior, diminished response to stimuli) and health disorders (gradual weight loss, tumor reached diameter of 12 mm or more) [44], the mice were humanely euthanized. At the conclusion of the experiment, all the mice were euthanized, and the tumors were excised and weighed. All animal studies were approved by the laboratory animal care committee of Xi’an Jiaotong University, and were performed according to the committee’s guidelines for the use of laboratory animals.

### 2.5. Hematoxylin and Eosin Staining

After 24 h fixation within 4% formaldehyde in PBS, the excised tumors were embedded in paraffin for sectioning. The tumor sections were adhered to the glass slides (pretreated with 0.01% aqueous solution of poly-L-lysine) and heated at 60 °C. After washing in xylene and rehydration through a graded series of ethanol, hematoxylin and eosin (H&E) staining of the tumor sections occurred according to the manufacturer’s protocol. The slides were then observed under microscopy (BX53; Olympus, Tokyo, Japan).

### 2.6. Biochemical Indicator Evaluation of Serum

Mouse blood was obtained from the heart using a draw-blood needle after sacrificing. Then, the blood was collected in a centrifuge tube and centrifuged at 3000 rpm for 15 min. The serum was collected for biochemical indicator detection (each group consisted of five mice, *n* = 5). All the serum samples were analyzed by Wuhan Seville Biological Technology Company.

### 2.7. Cell Viability Assays, Intracellular ROS Measurement, and Apoptosis Analysis

In this study, 5 × 103 T24 or J82 cells/well were seeded in 24-well plates for 30% saline or 30% PAS treatment. We replaced the mixed-medium (added 30% saline or PAS) every two days (three times a week). A Cell-Titer-Glo^®^ luminescent cell viability assay kit (Promega, Madison, WI, USA) was used to assess the viability of 30% saline and 30% PAS (the interval time between generating the PAS and addition to the cell culture is 1 h) treated cells following the manufacturer’s instructions. Intracellular ROS levels were measured with the ROS assay kit (Beyotime, Shanghai, China) following the manufacturer’s instructions. In brief, the sample cells were incubated with 10 µM DCFH-DA in medium at 37 °C for 25 min. Next, to remove the residual DCFH-DA fluorochrome, the cells were washed with PBS three times and then analyzed by flow cytometry (Accuri C6, BD Biosciences, New York city, NY, USA). For the apoptosis analysis, the cells were collected and washed three times with PBS and then suspended in 300 µL of 1 × Annexin V binding buffer containing 4 µL Annexin V-FITC and 4 µL PI. After 15 min incubation in the dark at 25 °C, cells were washed with 1 × Annexin V binding buffer for the following flow analysis. In addition, a Human Apoptosis Array Kit (R&D Systems, Minneapolis, MN, USA) was used to analyze the expression profiles of apoptosis-related proteins in PAS-treated cells [35]. All experiments were performed three times (*n* = 3).

### 2.8. Statistical Analysis

All data were presented as the mean ± SD. The Student’s *t*-test was applied to evaluate the statistical significance. *p* < 0.05 between two independent groups was considered statistically significant (* *p* < 0.05, ** *p* < 0.01).

## 3. Results

### 3.1. Surface Discharge Plasma and Aqueous Reactive Species Generation

Figure 1a illustrates the surface discharge plasma setup in this study. Figure 1b shows a photograph of the plasma device and plasma discharge. During direct irradiating of saline, surface discharge plasma is a rich source of ROS and RNS. The aqueous ROS and RNS concentrations increase with the plasma irradiation dose. As shown in Figure 1c,d, after plasma irradiation for 20 min, the hydrogen peroxide (H_2_O_2_), nitrite ion (NO_2_^−^) and nitrate ion (NO_3_^−^) concentrations in saline increased from 0.79 µM to 37.65 µM, 4.41 µM to 177.93 µM, and 3.25 µM to 130.36 µM, respectively. The concentration of spin trap adduct DMPO-OH in saline increased from 0.02 µM to3.85 µM (Figure 1e), and the concentration of TEMPONE increased from 0.04 µM to 48.43 µM after plasma irradiation for 20 min (Figure 1f). The increased DMPO-OH concentration indicates the increase of aqueous hydroxide radicals (OH•), as well as the increase in TEMPONE concentration indicating that the relative amounts of superoxide anion radicals (O_2_^−^) and peroxynitrite (ONOOH/ONOO^−^) increased. Therefore, it is considered that plasma activates the saline by generation of various ROS and RNS.

### 3.2. PAS Injection and In Vivo Anticancer Effects

**Figure 2:**
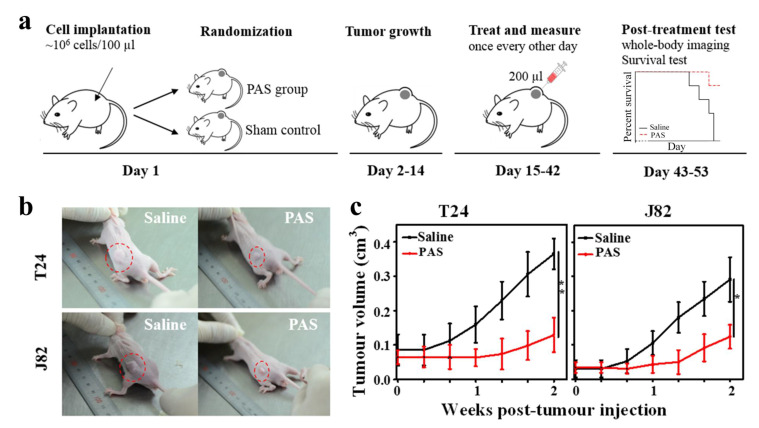
(**a**) Sketch map of saline and plasma-activated saline (PAS) injection in nude mice bearing tumors. (**b**) Typical image of mice with tumors for saline and PAS injection after 28 days. (**c**) Time-dependent changes in the tumor volume in xenograft models for saline and PAS injection after 14 days (*n* = 5) (* *p* < 0.05, ** *p* < 0.01).

To evaluate the potential of PAS in the treatment of MIBC, tumor models were established by subcutaneous injection of T24 and J82 cells expressing green fluorescent protein (GFP) into the left rear flank of male BALB/c male mice. Fourteen days after the injection with cancer cells, 10 mice bearing T24 tumors were randomly divided into two equal-sized groups: (1) tumor-bearing mice undergoing pericarcinomatous tissue injection with saline; (2) tumor-bearing mice undergoing pericarcinomatous tissue injection with PAS. Ten mice bearing J82 tumor underwent the same grouping mode for saline or PAS injection. As shown in Figure 2a, 200 µL saline or PAS (plasma activation for 20 min) were injected into a subcutaneous site near the tumor. The injection of saline and PAS was repeated three times a week. As shown in Figure 2b, at 14 days after saline or PAS injection, the in vivo experiments showed that the tumor with PAS injection was significantly smaller than the saline injection group, regardless of the cancer cell line. This means that the long-term pericarcinomatous tissue injections with PAS may help to prevent muscle-invasive bladder tumor growth. Figure 2c shows the tumor volume dependence on treatment time; after 14 days injection with saline, the volumes of the T24 and J82 tumors were increased to 0.37 cm^3^ and 0.29 cm^3^, respectively. For PAS injection, the volumes of the T24 and J82 tumors were increased to just 0.13 cm^3^, 0.12 cm^3^, respectively, after 14 days of treatment.

### 3.3. Cancer Metastasis and Survival Evaluation of PAS Therapy

**Figure 3:**
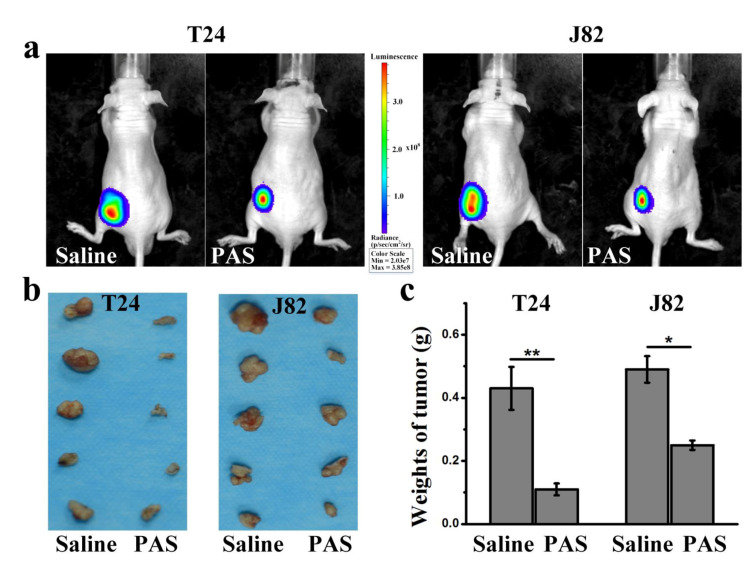
(**a**) The growth of bladder cancer cells shown by a whole-body fluorescent imaging system after saline and PAS injections. (**b**) Digital photographs of excised T24 and J82 tumors after saline and PAS injections. (**c**) Weight of excised tumors after different treatments (*n* = 5) (* *p* < 0.05, ** *p* < 0.01).

**Figure 4:**
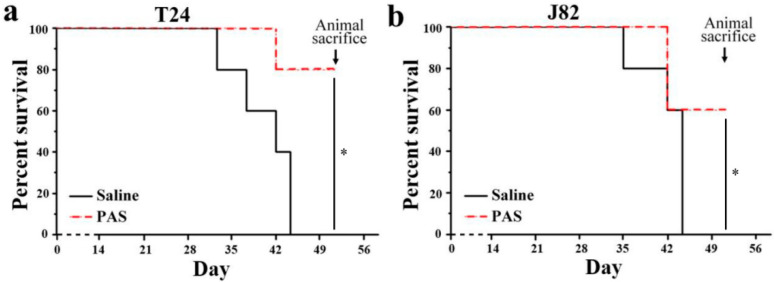
PAS treatment effect on survival of mice in a xenograft muscle-invasive bladder cancer (MIBC) model. (**a**) T24 and (**b**) J82 (*n* = 5) (* *p* < 0.05).

Next, the tumor bearing mice were assessed by in vivo fluorescence imaging to evaluate the MIBC migration after saline and PAS injection. As shown in Figure 3a, after 28 days of treatment, even though both the tumors with saline and PAS injection underwent no distant metastasis, the bioluminescence areas of the MIBC tumors with saline injection were about three times that with PAS injection, regardless of the cancer cell line. The relative volume and weight of the tumors from the PAS-injection mice were dramatically lower than that from the saline-injection mice (Figure 3b,c). On average, the weight of T24 tumors from the PAS-injection mice was only 25.6% of the tumor weight from the saline-injection mice; the weight of J82 tumors from the PAS-injection mice was around 53.1% of the tumor weight from the saline-injection mice. Furthermore, we also evaluated the survival of mice receiving saline and PAS injections. As shown in Figure 4a,b, there were significant differences in survival rate between the saline injection group and the PAS injection group. These results indicated the potential of PAS to inhibit muscle-invasive bladder tumor growth in vivo.

### 3.4. Biological Safety after PAS Injection

In order to examine the safety of PAS in vivo, histological analysis was performed on the organs (kidneys, liver, spleen, heart and lungs) resected from the mice. Tissues were harvested and stained with hematoxylin and eosin (H&E). Supplementary Figure S1 compares the tissue sections from the control (without tumor and liquid injection), saline injection, and PAS injection groups; no discernible toxicity was observed in PAS-treated mice. In addition, we investigated the blood biochemical indices, including liver function, kidney function, and myocardium enzymogram. As shown in Table 1, Table 2 and Table 3, the results indicate that PAS injection did not affect the blood biochemical index of the mice. All the results confirm that PAS (plasma activation for 20 min) is nontoxic and safe to use in vivo.

### 3.5. In Vitro Anticancer Effects and Mechanisms of PAS

**Figure 5:**
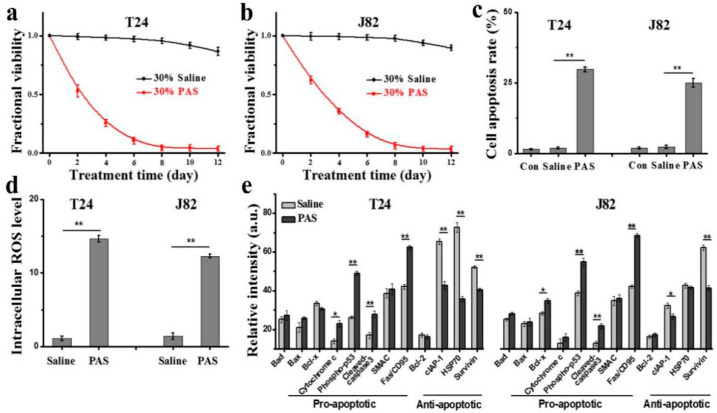
In vitro anticancer effects and mechanisms of PAS (**a**) and (**b**) treatment time-dependent changes in the cell viability of T24 and J82 cells after 30% saline and 30% PAS treatment (normalized to control); (**c**) apoptosis rates of T24 and J82 cells after 30% saline and 30% PAS treatment for 48 h; (**d**) intracellular ROS levels of T24 and J82 cells after 30% saline and 30% PAS treatment for 48 h (normalized to control); (**e**) cell apoptosis-related protein array analysis of T24 and J82 cells after 30% saline and 30% PAS treatment for 24 h (*n* = 3) (* *p* < 0.05, ** *p* < 0.01).

As excessive accumulation of plasma-generated ROS in cell culture medium can cause oxidative stress to cells, we investigated the cell viability, apoptosis rate, and intracellular ROS levels of T24 and J82 cells after culturing with 30% saline and 30% PAS. As with the in vivo injections, the addition of saline and PAS was repeated three times a week. As shown in Figure 5a,b, a CellTiter-Glo assay showed that, compared with the control group, the viability of T24 and J82 cells cultured with PAS gradually decreased in a treatment time-dependent manner, whereas the viability of the saline group was basically consistent with the control group. In addition, the apoptosis analysis results demonstrated that PAS can further induce T24 and J82 cancer cells apoptosis (Figure 5c). The statistical results of the flow cytometry indicated that PAS can enhance intracellular ROS levels regardless of the cancer cell line (Figure 5d). The intracellular ROS levels of T24 cells increased by about 14.6 times after culturing with 30% PAS for 48 h compared with the control, and the intracellular ROS levels of J82 cells increased about 12.2 times, while the intracellular ROS levels of T24 cells and J82 cells cultured with 30% saline did not rise. To elucidate the underlying mechanism of PAS-induced apoptosis, the expression of apoptosis-related proteins in T24 and J82 cells was assessed by using a human apoptosis protein array. As shown in Figure 5e, the expression levels of caspase 3 and Fas/CD95 were significantly increased in both T24 and J82 cells after the addition of 30% PAS for 48 h, while that of Survivin was decreased by PAS treatment. These results indicated that 30% PAS could efficiently induce the accumulation of intracellular ROS levels in T24 and J82 cells, and further trigger apoptosis through the activation of Fas/CD95 and downstream caspase cascades.

## 4. Discussion

Recently, CAP has attracted great interest in the field of cancer therapy. Previous studies have indicated that appropriate doses of direct CAP irradiation can effectively kill multiple types of cancer cells, with little cytotoxic effect on normal tissue cells, in vitro and in vivo. For example, Keidar et al. reported that CAP irradiation can induce apoptosis and the decrease of cell migration velocity in SW900 cancer cells, while leaving normal cells essentially unaffected [22]. Kang et al. indicated that direct CAP irradiation selectively reduced HNC cell viability in a dose-dependent manner, and triggered apoptosis by a mechanism involving MAPK-dependent mitochondrial ROS [23]. In addition, recent studies on mouse models with subcutaneous tumors and clinical patients have also validated the antitumor effects of CAP irradiation therapy [22,25,50,51,52]. The principal mode of interaction between plasma and cancer cells is considered to be the delivery of ROS and RNS. As we know, there are markedly different endogenous ROS levels between the cancer cells and normal cells. Due to hyperactive metabolism and rapid proliferation, cancer cells produce a lot of byproducts, such as ROS (hydrogen peroxide (H_2_O_2_), hydroxide radicals (OH•), superoxide anion radicals (O_2_^−^) and ozone (O_3_)), and this results in a higher level of endogenous ROS in cancer cells than in normal tissue cells [53,54]. Despite the free radical clear system, higher endogenous ROS levels make it more difficult for cancer cells to handle the excessive oxidative stress brought by additional ROS compared to normal tissue cells [55,56,57,58]. This makes it possible to selectively kill or inhibit the growth of cancer cells, while leaving normal cells intact, by supplying appropriate exogenous ROS. It has been extensively proven that there are types of reactive species (such as energy ions, excited molecules, free radicals, electric fields, and UV radiation) in the gas phase of CAP [59]. When CAP irradiates cells cultured in vitro or living tissues, the gas-phase reactive species can be trapped by the medium or blood surrounding the cells, where they then initiate a series of reactions to generate a large number of liquid-phase ROS and RNS, such as ozone (O_3_), hydrogen peroxide (H_2_O_2_), hydroxide radicals (OH•), superoxide anion radicals (O_2_^−^), nitrite ions (NO_2_^−^), nitrate ions (NO_3_^−^), peroxynitrite (ONOOH/ONOO^−^), and singlet oxygen (O_2_(^1^Δg)) [49,60,61,62]. These CAP-generated exogenous ROS and RNS can cause the accumulation of intracellular ROS levels, and eventually trigger programmed cell death.

Despite the promising anticancer effects, the action depth of direct CAP irradiation is severely restricted by the limitations of gas-phase reactive species delivery. As discussed above, CAP-induced selective anticancer effects depend on the ROS and RNS being produced and delivered to the biological target. Hence, the clinical adoption of direct CAP irradiation is limited to skin cancer and exposed tumor tissues. Although direct CAP irradiation therapy is not suitable for deep tumors and intracavity tumor targets, the application of plasma-activated solution could be an alternative therapy pathway [24]. Plasma-activated solution as an application mode of CAP has a very distinct superiority in reactive species storage and delivery. Based on this advantage, plasma-activated solution can break through the treatment-depth limitation of direct CAP irradiation and achieve deep tumor treatment. Especially for intracavity tumors like muscle-invasive bladder cancer, the soaking of the tumor tissue with plasma-activated solution may be a new therapeutic approach to substitute intravesical perfusion with chemotherapeutics. In addition, considering the high selectivity and low side effect profile, plasma-activated solution therapy may be quite suitable as a daily treatment.

In this study, we demonstrated the feasibility of PAS as a local medicament for muscle-invasive bladder cancer treatment. The in vivo experiments showed that PAS efficiently introduces an antiproliferative effect on both T24 and J82 tumors. The results of pathological slices and serologic analysis indicated that long-term administration of PAS does not cause any toxic or side effects to nude mice. Recently, the anticancer effects were observed when the cells were treated with a plasma jet-activated medium in vitro [41,49,63], and even antitumor effects in vivo [64,65,66,67]. For other plasma-activated liquids, it has been reported that Ringer’s lactate solution and phosphate-buffered saline solution also exhibit significant anticancer effects in vitro and in vivo through activation by plasma jet [16,68]. These authors speculated that the mechanism of these anticancer effects was related to oxidization by plasma jet-generated aqueous ROS and RNS. Our results also confirmed the high oxidation activity of surface discharge plasma-activated saline. While we did not show the antitumor effects of PAS by intravesical perfusion for orthotopic muscle-invasive bladder cancer, our results also demonstrated the analogical antitumor effects by the soaking of the tumor tissues with PAS through pericarcinomatous tissue injection.

To elucidate the underlying mechanism of PAS-induced antitumor effects on T24 and J82 bladder cancer, we further studied the effects of PAS on T24 and J82 cells in vitro. Our results revealed that the addition of 30% PAS in medium can inhibit the viability of both T24 and J82 cells through enhancing the intracellular ROS level, and trigger cancer cell apoptosis by activating the ROS-mediated Fas/CD95 pathway. Several previous studies have indicated that plasma-generated aqueous ROS and RNS can cause mitochondrial dysfunction, DNA damage, and cell cycle arrest, and further induce cancer cell apoptosis, autophagy, or necrosis [23,41,42,43]. Indeed, the abnormal intracellular ROS levels can be utilized as a cytotoxic mechanism by the innate immune systems of cells [69]. In addition, among various aqueous ROS and RNS generated by CAP, both the long-lived reactive species (such as hydrogen peroxide (H_2_O_2_) and nitrate ions (NO_3_^−^)) and the short-lived reactive species (such as hydroxide radicals (OH•), superoxide anion radicals (O_2_^−^), and peroxynitrite (ONOOH/ONOO^−^)) are proposed to play major roles in the anticancer effects [13,38].

Although further studies are needed, our initial results demonstrated the feasibility of PAS therapy as a novel treatment option for muscle-invasive bladder cancer by both in vitro and in vivo experiments. Considering the promising anticancer effects and minimal side effects, paracancerous injection or intravesical perfusion with PAS for local MIBC treatment, just like intravesical chemotherapy, is likely to become an achievable clinical treatment technique.

## 5. Conclusions

In this study, we evaluated the feasibility of PAS for muscle-invasive bladder cancer (MIBC) therapy both in vivo and in vitro. Our data showed that pericarcinomatous tissue injection with PAS is effective at preventing muscle-invasive bladder tumor (T24 and J82 cell lines) growth, with no side effects, in nude mice after long-term administration. CAP-generated aqueous ROS and RNS may be essential for Fas/CD95-mediated cell apoptosis in response to PAS treatment. In terms of MIBC therapy in the future, we recommend intravesical perfusion with PAS as a promising treatment option, although further studies on the clinical antitumor effects and the involved mechanisms are essential.

## Figures and Tables

**Table 1:** Analysis of liver function after PAS treatment.

Indicator	Control	Saline	PAS
Alkaline phosphatase (U/L)	125.43 ± 1.75	122.161 ± 0.81	124.186 ± 2.20
Glutamate aminotransferase (U/L)	3.655 ± 0.19	3.205 ± 0.85	3.440 ± 0.48
Glutamic-pyruvic transaminase (U/L)	45.623 ± 3.91	45.815 ± 1.52	42.569 ± 2.46

**Table 2:** Analysis of kidney function after PAS treatment.

Indicator	Control	Saline	PAS
Urea/Urea nitrogen (mg/dl)	28.724 ± 1.04	30.104 ± 4.33	26.918 ± 1.81
Creatinine (µmol/L)	40.402 ± 4.12	41.093 ± 4.34	41.712 ± 1.04
Uric acid (µmol/L)	168.466 ± 7.63	171.596 ± 9.87	177.778 ± 2.36

**Table 3:** Analysis of myocardium enzymogram after PAS treatment.

Indicator	Control	Saline	PAS
Creatine kinase (U/L)	4939.857 ± 34.95	4974.272 ± 168.42	5713.316 ± 86.24
Lactate dehydrogenase L (U/L)	1417.425 ± 108.75	1419.608 ± 61.02	1479.396 ± 191.23
Lactate dehydrogenase isozyme (U/L)	66.480 ± 5.25	64.943 ± 2.46	63.38967 ± 6.58

## Data Availability

No new data were created or analyzed in this study. Data sharing is not applicable to this article.

## Supplementary Materials

The following are available online at https://www.mdpi.com/2072-6694/13/5/1042/s1, Figure S1: Representative histological H&E stained tissue sections of mice organ slices after different treatments. All images share the same scale bar of 50 μm.
