# Supplementary material for: The Antitumor Effects of Plasma-Activated Saline on Muscle-Invasive Bladder Cancer Cells In Vitro and In Vivo Demonstrate Its Feasibility as a Potential Therapeutic Approach
Source: Cancers (Basel). 2021 Mar 2;13(5):1042. doi: 10.3390/cancers13051042 (PMC7958317; doi:10.3390/cancers13051042)
Supplement: Supplementary file 1 [file cancers-13-01042-s001.pdf]

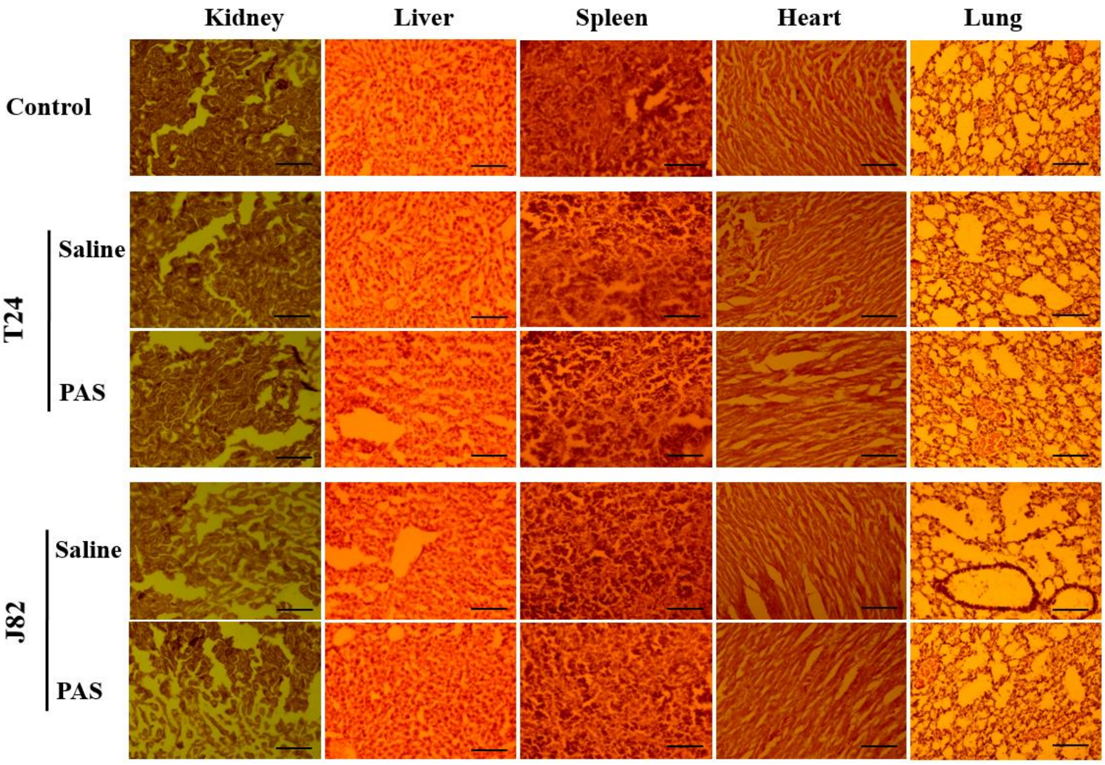

Supplementary Figure 1. Representative histological H&E stained tissue sections of mice organ slices after different treatments. All images share the same scale bar of 50 μm.
